# Applying the Moving Epidemic Method to Emergency Department Syndromic Surveillance to identify the Start of Influenza Seasons in New South Wales, Australia

**DOI:** 10.1111/irv.70293

**Published:** 2026-07-15

**Authors:** Nectarios Rose, Adam T. Craig, David J. Muscatello

**Affiliations:** ^1^ University of New South Wales Sydney Australia; ^2^ The University of Queensland Australia

**Keywords:** emergency department syndromic surveillance, influenza, MEM, moving epidemic method, thresholds

## Abstract

**Background:**

The Moving Epidemic Method (MEM) is a widely used threshold‐based approach for identifying the start of an influenza season but has rarely been used with emergency department (ED) syndromic surveillance (EDSyS). This retrospective study aimed to evaluate MEM applied to rates of ED presentation for conditions compatible with influenza to calculate thresholds for identifying the start of the influenza season (pre‐epidemic thresholds) in New South Wales, Australia, 2012 through 2019.

**Methods:**

MEM was tested on weekly rates of ED presentations for three Syndromic Groups based on combinations of four diagnostic syndromes related to influenza, to calculate pre‐epidemic thresholds (epidemic start threshold) for the years 2012–2019. Validation included comparing the start of a season using pre‐epidemic thresholds with the start of the season based on percent positive influenza notifications from sentinel laboratories (identification difference).

**Results:**

The most accurate model for identifying the start of the influenza season combined all four diagnostic syndromes, consisting of influenza like illness, unspecified viral illness, pneumonia and lower respiratory tract infections. Pre‐epidemic thresholds based on MEM were able to identify the influenza season from between 2 weeks before to 1 week after the start, with a mean of 0.6 weeks before the start of a season and a mean absolute identification difference of 1.1 weeks.

**Conclusion:**

This study demonstrates the utility of MEM with EDSyS for identifying the start of influenza seasons, providing a visual alert for optimal timing of public health preparedness and response.

## 1 Introduction

Surveillance systems that monitor seasonal influenza activity are critical for optimising the timing of interventions that minimise transmission in the community, reduce absenteeism, improve health outcomes and reduce the impact on health services [1]. Designing and validating surveillance systems is complicated by the lack of accepted criteria to define the exact start of a season. Furthermore, seasonal influenza activity can vary significantly in timing, magnitude and duration from year to year, posing a challenge to developing accurate systems for predicting and identifying season onset. For example, the influenza season in New South Wales (NSW)—the largest and most populous state in Australia (~8.1 million in 2019)—typically starts in April and ends by October, with seasons beginning as late as July [2, 3].

Public health surveillance systems rely on various data sources to monitor influenza activity, including counts and percent positivity of laboratory influenza notifications (percent positivity), rates of medically attended acute respiratory infections (ARI) and influenza like illness (ILI), community surveys and even social media activity [4, 5, 6]. Emergency department (ED) syndromic surveillance (EDSyS) systems use preliminary diagnosis based on symptoms and clinical signs, offering the advantage of speed and relative cost‐efficiency, while being as sensitive in identifying seasonal influenza onset as surveillance based on confirmatory testing [7, 8]. EDSyS systems generally employ statistical aberration detection methods such as cumulative sum‐based (CUSUM) methods, scan statistics, exponentially weighted moving averages (EWMA), change‐point detection and statistical control charts [9, 10, 11, 12]. These approaches work by identifying a greater frequency of cases than expected compared to previous seasons, or where the rate of increase in activity exceeds some pre‐defined threshold. Threshold‐based techniques rely on historical surveillance data to identify when real‐time data crosses a calculated epidemic threshold.

The Moving Epidemic Method (MEM) is a widely used threshold‐based approach [13] for identifying the start of an influenza season that is not commonly used with EDSyS despite its widespread application to ILI [14, 15, 16, 17, 18] and acute respiratory infection (ARI) presentations [19, 20] in outpatient/GP presentations, virology data [21, 22], ILI related claims data [23], influenza related hospitalisations and deaths [17] and oseltamivir prescriptions [24]. An Australian study explored the use of MEM with data from survey‐based weekly ILI rates, public health hotline inquiries and ILI visits to GPs [18] but MEM has not been applied to EDSyS in Australia.

This retrospective study aimed to assess the use of MEM on rates of ED presentation for conditions compatible with influenza to develop thresholds for identifying the start of the influenza season.

## 2 Methods

### 2.1 Data

The study analysed data extracted from the NSW statewide de‐identified Pandemic and Epidemic Assessment of Risk Linked data (PEARL) Database. PEARL contains individually linked, anonymised health records of people presenting to NSW EDs with a condition compatible with ARI for years 2005 (78 EDs) through to 2022 (169 EDs) [25]. To consolidate records across different EDs and codesets used for assigning a primary diagnosis (ICD9, ICD10 and SNOMED CT), ED diagnosis codes that indicated influenza or other non‐specific ARI were mapped to 16 categories that we term ‘ED Syndromes’. Previous work [26] found that in addition to the influenza specific ED Syndromes—ILI and Influenza—Unspecified Viral also had high correlation and similarity measures when compared to percent positivity. Given their high rates of admission and confirmatory laboratory diagnosis for influenza, LRTI and Pneumonia were also recommended for use in EDSyS. The definition of these ED syndromes is described in (Table 1).

**TABLE 1:** ED diagnosis inclusion criteria for ED syndromes.

ED syndrome	Inclusion criteria^a^
ILI/Influenza	Influenza: Influenza, including secondary condition caused by influenza OR ILI: Specific mention of influenza like illness
Unspecified viral	Non‐specific viral illness, without reference to the respiratory system
LRTI	Lower respiratory tract infection, respiratory infection or viral pneumonia
Pneumonia	Pneumonia due to unknown, unspecified bacteria or *S pneumoniae, S aureus, H influenzae*

^a^ ICD9, ICD10 and SNOMED CT inclusions can be found in [26].

### 2.2 Analysis

Time series of ED presentation rates for each syndrome per 1000 total ED presentations by epiweek were prepared. Based on the findings from our previous work [26], we tested three combinations of ED Syndromes or ‘Syndromic Groups’: ILI and Influenza (Syndromic Group 1); ILI, Influenza and Unspecified viral (Syndromic Group 2); and ILI, Influenza, Unspecified viral, Pneumonia and LRTI (Syndromic Group 3). Descriptive analysis included visualising each time series and calculating relative rate ratios by week for Syndromic Group 2 and 3 compared to Syndromic Group 1.

The MEM was applied to each of these time series to calculate the pre‐epidemic threshold—the rate of ED presentations which signals the start of the influenza season. There are three steps in the MEM algorithm which have been described in detail elsewhere [13, 19, 27], with the first two being applicable to this study. In step 1 of MEM, the Maximum Accumulated Percentage (MAP) is used to identify the most active part of a season by determining the minimum number of consecutive weeks that account for the highest cumulative percentage of rates. There are two approaches described in the literature for calculating the optimum consecutive number of weeks using the MAP. The first is based on the original method described by Vega et al. (2004) [27] and involves plotting the derivative of the MAP after first smoothing the original data to avoid irregularities and then calculating the inflection point of the resulting curve. A second method, known as the fixed criterium method, uses the slope of the MAP curve when it exceeds a predefined value delta [19]. The data may also be smoothed beforehand, typically using a moving average of 3 weeks [13]. We tested and compared both calculation approaches in this study, with and without smoothing for the fixed criterium method. Step 2 of MEM involves calculating the epidemic threshold by determining the upper limit of the 95% one‐sided confidence interval of a set of highest pre‐epidemic weekly rates from historical seasons.

PEARL data from 2006 was used to provide the recommended minimum of 6 years of historical data required to calculate pre‐epidemic thresholds for each of the target years 2012–2019 [13, 28]. The years 2020–2022 were omitted from this study due to the disruption caused by the COVID‐19 pandemic.

### 2.3 Validation

The MEM package in R employs cross‐validation where each season is treated as the target season, and other seasons are used as the historical seasons. This allows the calculation of sensitivity and specificity by comparing epidemic and non‐epidemic periods of the target and historical epidemic seasons. We initially tested the range of delta values recommended in the MEM package—from 2.0 to 4.0 at increments of 0.1—with the final selected value based on the highest Youden index, calculated as the sum of sensitivity and specificity minus one.

To validate the models against real world observations, each calculated pre‐epidemic threshold was used to determine when it would have crossed the ED time‐series of the target season for at least two consecutive weeks. We refer to this epiweek as the pre‐epidemic threshold based season start. As a comparative reference for the start of each season, we identified the epiweek which saw the beginning of a sustained increase in the percent positivity from sentinel laboratories in NSW, which account for > 80% of all NSW influenza notifications [29]. The difference between the pre‐epidemic threshold‐based season start and the percent positivity‐based season start, which we termed the identification difference (ID), was calculated for each model, with mean absolute identification difference (MAID) providing an assessment of average model accuracy across all target seasons. A negative ID value means MEM identified a season before the start based on percent positivity.

Data analysis was carried out using SAS/STAT software, Version 9 of the SAS System for Windows. We implemented our models with the original method and fixed criterion method using the MEM package (version 2.19) in R [30].

## 3 Results

### 3.1 Descriptive Analysis

**FIGURE 1:**
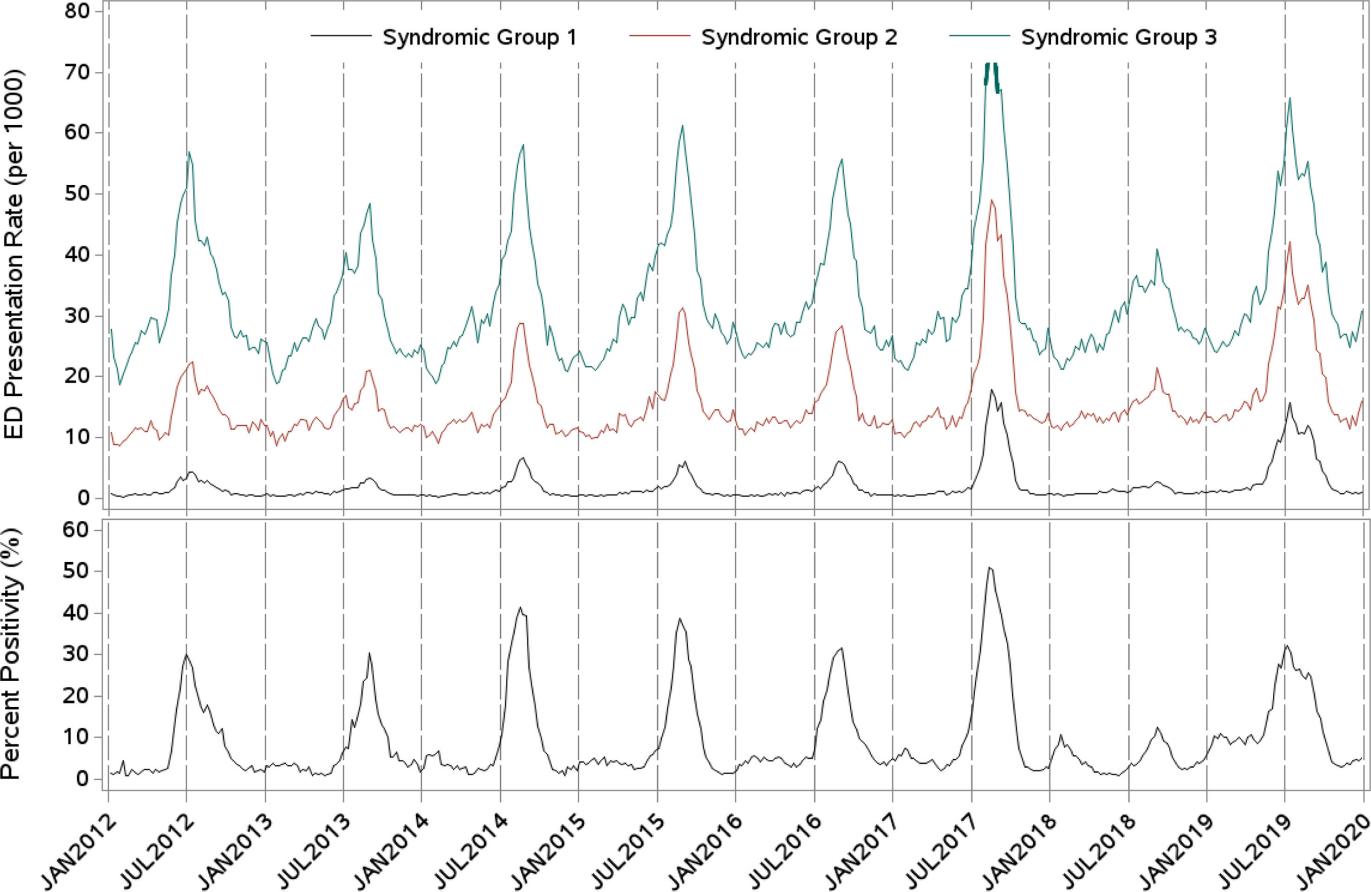
Weekly time series of ED presentation rates for the three Syndromic Groups (top) and percent positivity of influenza notifications (bottom).

Across all three Syndromic Groups, the patterns in syndromic activity (i.e., the peaks and troughs) are similar. Of these, Syndromic Group 2 and 3 showed greater activity in the months before the start of each influenza season (Figure 1). Syndromic Group 3 presentation rates on average were 33 times the rates of Syndromic Group 1 for the equivalent epiweek per 1000 presentations (quartiles 1–3: 4–97), while Syndromic Group 2 presentations were 15 times the rates of Syndromic Group 1 (quartiles 1–3: 2–21).

Table 2 shows the overall performance of each Syndromic Group across the years 2012–2019 comparing the original and fixed criterium methods (without initial smoothing). The mean Youden index for Syndromic Group 1 was highest for both methods. However, Syndromic Group 3 had the lowest MAID. Using the original method with Syndromic Group 3 returned a MAID of 1.1 weeks with an ID range of −2 to 1 weeks, compared to the fixed criterium method of 2.4 weeks and a range of −5 to 7 weeks. Syndromic Group 3 performance measures did not improve when a 3‐week moving average smoothing was applied to the raw data, while performance measures were worse for Syndromic Groups 1 and 2 (Tables S1 and S2).

**TABLE 2:** Performance of each model based on Step 1 method in MEM used for years 2012–2019.

Method	Syndromic group	ID (Min)	ID (Max)	Mean ID	MAID	Youden (mean)
Fixed criterium	1	−2	10	4.4	4.9	0.69
Fixed criterium	2	−5	7	2.6	3.9	0.33
Fixed criterium	3	−5	7	0.4	2.4	0.33
Original	1	−8	9	1.5	5.5	0.67
Original	2	−5	2	0.2	1.9	0.32
Original	3	−2	1	−0.6	1.1	0.33

Abbreviations: ID: Identification difference; MAID: Mean absolute identification difference.

Across all seasons tested for Syndromic Group 3, the original method performed consistently better than the fixed criterium method (Table 3). The fixed criterium method performed poorly in 2018 and 2019, identifying the season 7 weeks after and 5 weeks before the start, respectively. In comparison, the original method identified the season 2 weeks before the start for the same years. After finding optimal delta values of 2.0 for four of the seasons using the fixed criterium method, we extended the lower range of values tested down to 1.6 at which point the MEM package in R failed. The failure of the MEM package was identified by the developers to be a coding issue and due to be fixed in a future release. The final optimal delta values for Syndromic Group 3 ranged from 1.6 to 2.3.

**TABLE 3:** Performance of MEM for Syndromic Group 3 for each year between 2012 and 2019 based on method used for Step 1 in MEM.

Method	Year	Delta	Percent positivity based start	Pre‐epidemic threshold based start	Identification difference
Fixed criterium	2012	2.1	20	21	1
Fixed criterium	2013	2.1	24	23	−1
Fixed criterium	2014	1.9	24	24	0
Fixed criterium	2015	2	21	19	−2
Fixed criterium	2016	2.2	25	27	2
Fixed criterium	2017	2	22	23	1
Fixed criterium	2018	2.3	29	36	7
Fixed criterium	2019	1.6	18	13	−5
Original	2012	N/A	20	20	0
Original	2013	N/A	24	23	−1
Original	2014	N/A	24	24	0
Original	2015	N/A	21	19	−2
Original	2016	N/A	25	26	1
Original	2017	N/A	22	23	1
Original	2018	N/A	29	27	−2
Original	2019	N/A	18	16	−2

**FIGURE 2:**
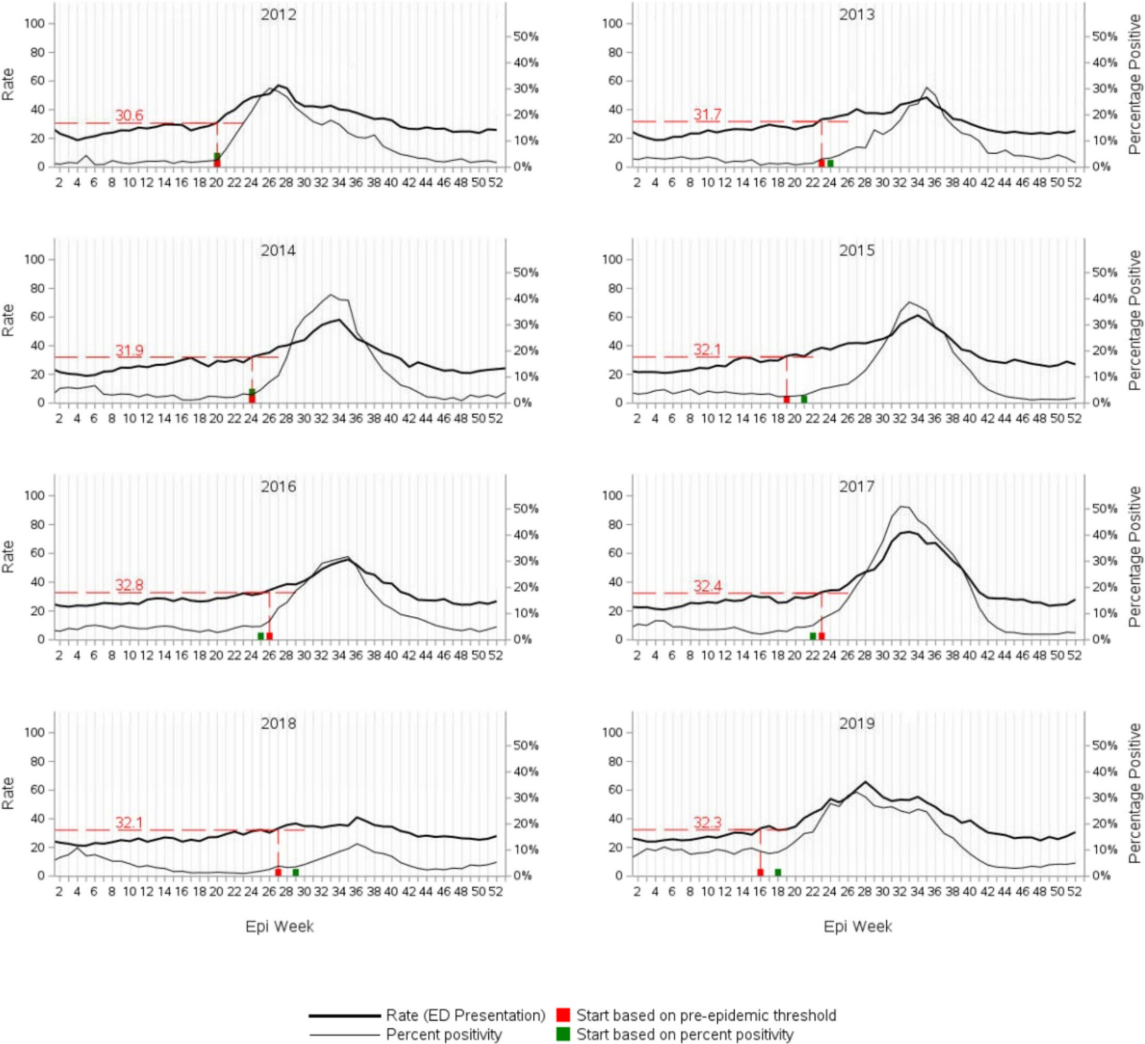
Time series of Model 3 (left y‐axis) and percent positivity influenza notifications (right y‐axis) by epiweek, showing MEM pre‐epidemic thresholds based on six historical seasons and the corresponding epiweek where the pre‐epidemic threshold is crossed (red). The start of each season based on percent positivity is shown as green.

Figure 2 shows the time series for the best model based on MAID (Syndromic Group 3 using the original method), the pre‐epidemic threshold value and the epiweek for the start of the season based on the pre‐epidemic threshold. Also included in the figure is the time series of the corresponding percent positivity for that year, and the start of the season based on percent positivity. The pre‐epidemic threshold ranged from a value of 30.6–32.8 ED visits per 1000. For most years, we see a transient spike in ED rates approaching the pre‐epidemic threshold before crossing it.

When averaged across the 2012–2019 seasons, a delta value of 2.0 had the highest Youden index and lowest MAID, with an ID of between −2 and 2 weeks (Table 4). Delta values between 1.7 and 2.3 also show relatively high Youden index and low MAID values, with delta values 1.8, 1.9 and 2.0 showing comparable results to the original method.

**TABLE 4:** Summary statistics for individual delta values tested with the fixed criterium method for Syndromic Group 3 across 2012–2019.

Delta	ID (Min)	ID (Max)	Mean ID	MAID	Youden (mean)
1.6	−13	−5	−9.3	9.3	0.26
1.7	−7	1	−2.4	2.6	0.32
1.8	−2	1	−0.8	1.0	0.32
1.9	−2	2	−0.1	1.1	0.33
2.0	−2	2	0.0	1.0	0.33
2.1	−2	7	1.5	2.3	0.32
2.2	1	7	2.5	2.5	0.32
2.3	1	7	2.9	2.9	0.31
2.4	−28	5	−0.8	6.3	0.29
2.5	−28	6	−0.3	6.8	0.27

Abbreviations: ID: Identification difference; MAID: Mean absolute identification difference.

## 4 Discussion

This study demonstrates the utility of applying MEM to identify the start of seasonal influenza using surveillance data based on influenza‐related ED presentations. Our results show that MEM, applied to weekly rates of ED presentations combining syndromes based on influenza and ILI, unspecified viral illnesses, lower respiratory tract infections and pneumonia can identify the start of the influenza season within an average of 1 week of the start based on percent positive influenza notifications. While a study in Mauritius applied MEM to ARI presentations associated with emergency departments, this was in the context of outpatient visits [31], and other hospital based studies have applied MEM using data based on admissions [21, 32, 33, 34] and outpatient presentations [22, 35]. Our study is unique in that it applies MEM in the context of an ED EDSyS system, which includes acute presentations for ARIs requiring admission, as well as less severe cases that can be discharged on the same day [36, 37].

Although the original method for Step 1 of MEM was first proposed in 2004 [27], experience with European data led to a preference for the fixed criterium method [19, 20], and only studies using this method have been published since [18, 21, 32, 33, 34, 38, 39, 40, 41]. This study directly compared the two approaches for Step 1 of MEM, demonstrating the accuracy of the original method in identifying the influenza season without the need to test and select delta parameter values. Given the original method includes a process for smoothing the raw data to avoid irregularities, we also applied smoothing to the fixed criterium method but found no improvement in performance measures. Studies that included details of the delta value selection used either the default value of 2.8 or tested values between the recommended range of 2.0–4.0 [22, 33, 38, 40, 41]. Our testing indicated that lower values than 2.0 produced better performance based on the Youden index. Our study also demonstrated that using the results of the target 2012–2019 seasons to retrospectively calculate a single delta value based on the highest average Youden index resulted in better performance than applying different delta values to each season. The disadvantage of selecting a delta value this way is that it is dependent on having at least one set of historic years and a corresponding target year, with several years preferable to improve the validity of the selection. While there are a number of optimisation options possible in the MEM R package [13, 28], our findings suggest that selection of the optimal delta value is the main factor affecting the performance of the fixed criterium method.

Threshold‐based approaches such as MEM are an alternative to aberration statistical techniques for EDSyS. In NSW, monitoring of influenza‐related activity includes the use of the Public Health Rapid Emergency Disease Surveillance System (PHREDSS), an EDSyS system that monitors for seasonal increases in ILI and pneumonia presentations [42]. PHREDSS applies a modified CUSUM‐based algorithm known as the index of increase to inform public health response to the start of a season, based on ED presentation counts exceeding a pre‐defined threshold [43]. MEM offers the advantage that it is a standardised and widely used and understood approach, and the concept of comparing current disease surveillance data against historical, seasonal patterns is intuitive [19]. A threshold also provides a visual alert in the context of current ED activity, allowing greater lead time to prepare for the start of a season as activity approaches the threshold. This situational awareness is useful in providing a better understanding of unseasonal activity compared with a seasonal outbreak. We note the transient increases in ED rates approaching the pre‐epidemic threshold (Figure 2), potentially leading to false alerts. We therefore recommend MEM be used and interpreted in conjunction with aberration techniques and other surveillance indicators to avoid false alerts.

Syndromic Group 3, which combined the ED Syndromes ILI/Influenza, Unspecified Viral, LRTI and Pneumonia, had the best performance in identifying the observed onset of the influenza season based on percent positivity. On average across all seasons, it identified the onset of influenza to within 1 week of the observed onset. It ranked second in terms of the Youden index, which was relatively low at 0.33 compared to the Youden index of ILI/Influenza at over 0.67 and 0.69, depending on the method used for Step 1 of MEM. This demonstrates that the cross‐validation metric used in the MEM R package should not be relied upon to predict real‐world performance.

The identification of the ‘true’ start of each season relies on the visual interpretation of each percent positivity time‐series. In NSW, there is no widely accepted approach to identifying the start of a season, with public health relying on review of the index of increase from PHREDSS data, percent positivity and other data sources using active and passive surveillance systems [5, 29]. Real‐time assessment of these data sources is described in weekly surveillance reports [3], but there has not been a retrospective review of the 2012–2019 years to consolidate these data and define exactly when the start of a season occurred. We used a pragmatic approach by identifying the earliest point where sustained, increasing transmission of influenza in the community has occurred, and therefore when public health action could have been best timed without triggering a false alert. To this end, percent positivity of laboratory notifications can identify the timing of a sustained increase in influenza cases more reliably than raw counts of influenza positive cases by accounting for the number of tests performed. Percent positivity has limitations, however, as an indicator for influenza incidence and infection levels as it is prone to bias by fluctuations in testing and infection rates [44]. Given this limitation, using a threshold with percent positivity to identify the start of a season is unreliable, with no empirical evidence to support the 5% threshold used in some studies [45, 46]. This is supported by a visual examination of the 2019 season in Figure 2 which shows inter‐seasonal activity of greater than 5% leading up to the start of the season. Here, we point out the distinction between techniques looking at real‐time data to identify the likely start date, compared to review of data retrospectively to assess when a season actually began. We believe visually examining the percent positivity time‐series in its entirety allows us to identify a consistent and sustained upward trend that marks the beginning of a season more reliably, without needing to implement statistical algorithms based on the same logic.

A limitation of this study is that the development and testing of models was carried out on seasons prior to the COVID‐19 pandemic. There was almost no influenza activity in 2020 and 2021, with a more typical season returning in 2022—albeit with a very early onset at around 12 weeks and with 2025 showing an unusually prolonged season [2, 3]. The impact of circulating COVID‐19 on the suitability of using ED Syndrome definitions for influenza is unclear and needs to be tested. The challenge is the availability of only three typical influenza seasons since COVID as historical seasons for MEM. This study suggests that specific ED Syndromes that define influenza do not work well with the MEM algorithm in the context of EDSyS based data, given MEM only performed well when unspecified viral, pneumonia and LRTI were included. Nevertheless, our study demonstrates that with the appropriate selection of ED presenting diagnoses and model parameters, MEM can be effective in determining pre‐epidemic thresholds in the context of EDSyS.

## 5 Conclusion

This retrospective study assessed the application of MEM with ED presentation rates for the years 2012–2019 in NSW, Australia, finding pre‐epidemic thresholds closely aligned with the start of increasing influenza transmission. Models which combined syndromes based on influenza and ILI, unspecified viral illnesses, lower respiratory tract infections and pneumonia performed best compared to syndromes based on just influenza and ILI. The original MEM method (plotting the derivative of the maximum accumulated percentage curve and calculating the inflection point) was simpler to implement and could be considered along with the fixed criterium method when implementing MEM.

## Author Contributions

**Nectarios Rose:** conceptualization, data curation, formal analysis, methodology, software, validation, visualization, writing – original draft, writing – review and editing. **Adam T. Craig:** supervision, writing – review and editing. **David J. Muscatello:** funding acquisition, supervision, writing – review and editing.

## Funding

This work was supported by the National Health and Medical Research Council (APP1194109).

## Conflicts of Interest

The authors declare no conflicts of interest.

## Supporting information


**Table S1:** Performance of fixed criterium method with a 3‐week moving average smoothing applied for years 2012–2019. ID: Identification difference. MAID: Mean absolute identification difference.
**Table S2:** Performance of fixed criterium method for ED Syndromic Group 3 with a 3‐week moving average smoothing applied for each year between 2012 and 2019.

## Data Availability

The data that support the findings of this study are available from The Centre for Health Record Linkage (CHeReL). Restrictions apply to the availability of these data, which were used under license for this study. Data are available from https://www.Cherel.org.au/ with the permission of the New South Wales Ministry of Health.
