# Supplementary material for: Applying the Moving Epidemic Method to Emergency Department Syndromic Surveillance to identify the Start of Influenza Seasons in New South Wales, Australia
Source: Influenza Other Respir Viruses. 2026 Jul 15;20(7):e70293. doi: 10.1111/irv.70293 (PMC13372569; doi:10.1111/irv.70293)
Supplement: Supplementary file 1 — Table S1: Performance of fixed criterium method with a 3‐week moving average smoothing applied for years 2012–2019. ID: Identification difference. MAID: Mean absolute identification difference. Table S2: Performance of fixed criterium method for ED Syndromic Group 3 with a 3‐week moving average smoothing applied for each year between 2012 and 2019. [file IRV-20-e70293-s001.docx]

**Supplementary file for:**

**Applying the Moving Epidemic Method to Emergency Department Syndromic Surveillance to identify the start of influenza seasons in New South Wales, Australia**

Nectarios Rose1, Adam T Craig2, David J Muscatello1

Table S1: Performance of fixed criterium method with a 3 week moving average smoothing applied for years 2012-2019.

ID: Identification difference. MAID: Mean absolute identification difference

| **Syndromic Group** | **ID (Min)** | **ID (Max)** | **Mean ID** | **MAID** | **Youden (mean)** |
| --- | --- | --- | --- | --- | --- |
| 1 | -28 | 9 | 0.5 | 8.0 | 0.69 |
| 2 | -28 | 4 | -1.8 | 6.5 | 0.33 |
| 3 | -4 | 7 | 1.5 | 2.5 | 0.33 |

Table S2: Performance of fixed criterium method for ED Syndromic Group 3 with a 3 week moving average smoothing applied for each year between 2012 to 2019.

| **Year** | **Delta** | **Percent Positivity based start** | **Pre-epidemic threshold based start** | **Identification Difference** |
| --- | --- | --- | --- | --- |
| 2012 | 2.2 | 20 | 22 | 2 |
| 2013 | 2.2 | 24 | 25 | 1 |
| 2014 | 2.0 | 24 | 25 | 1 |
| 2015 | 2.1 | 21 | 21 | 0 |
| 2016 | 2.3 | 25 | 28 | 3 |
| 2017 | 2.0 | 22 | 24 | 2 |
| 2018 | 2.2 | 29 | 36 | 7 |
| 2019 | 1.7 | 18 | 14 | -4 |
